# A simulation study for comparing testing statistics in response-adaptive randomization

**DOI:** 10.1186/1471-2288-10-48

**Published:** 2010-06-05

**Authors:** Xuemin Gu, J Jack Lee

**Affiliations:** 1 Department of Biostatistics, Division of Quantitative Sciences, The University of Texas MD Anderson Cancer Center, PO Box 301402, Unit 1409, Houston, Texas 77230-1402, USA; 2 Department of Biostatistics, Division of Quantitative Sciences, The University of Texas MD Anderson Cancer Center, PO Box 301402, Unit 1411, Houston, Texas 77230-1402, USA

## Abstract

**Background:**

Response-adaptive randomizations are able to assign more patients in a comparative clinical trial to the tentatively better treatment. However, due to the adaptation in patient allocation, the samples to be compared are no longer independent. At large sample sizes, many asymptotic properties of test statistics derived for independent sample comparison are still applicable in adaptive randomization provided that the patient allocation ratio converges to an appropriate target asymptotically. However, the small sample properties of commonly used test statistics in response-adaptive randomization are not fully studied.

**Methods:**

Simulations are systematically conducted to characterize the statistical properties of eight test statistics in six response-adaptive randomization methods at six allocation targets with sample sizes ranging from 20 to 200. Since adaptive randomization is usually not recommended for sample size less than 30, the present paper focuses on the case with a sample of 30 to give general recommendations with regard to test statistics for contingency tables in response-adaptive randomization at small sample sizes.

**Results:**

Among all asymptotic test statistics, the Cook's correction to chi-square test (*T*_*MC*_) is the best in attaining the nominal size of hypothesis test. The William's correction to log-likelihood ratio test (*T*_*ML*_) gives slightly inflated type I error and higher power as compared with *T*_*MC*_, but it is more robust against the unbalance in patient allocation. *T*_*MC *_and *T*_*ML *_are usually the two test statistics with the highest power in different simulation scenarios. When focusing on *T*_*MC *_and *T*_*ML*_, the generalized drop-the-loser urn (GDL) and sequential estimation-adjusted urn (SEU) have the best ability to attain the correct size of hypothesis test respectively. Among all sequential methods that can target different allocation ratios, GDL has the lowest variation and the highest overall power at all allocation ratios. The performance of different adaptive randomization methods and test statistics also depends on allocation targets. At the limiting allocation ratio of drop-the-loser (DL) and randomized play-the-winner (RPW) urn, DL outperforms all other methods including GDL. When comparing the power of test statistics in the same randomization method but at different allocation targets, the powers of log-likelihood-ratio, log-relative-risk, log-odds-ratio, Wald-type Z, and chi-square test statistics are maximized at their corresponding optimal allocation ratios for power. Except for the optimal allocation target for log-relative-risk, the other four optimal targets could assign more patients to the worse arm in some simulation scenarios. Another optimal allocation target, *R*_*RSIHR*_, proposed by Rosenberger and Sriram (*Journal of Statistical Planning and Inference*, 1997) is aimed at minimizing the number of failures at fixed power using Wald-type Z test statistics. Among allocation ratios that always assign more patients to the better treatment, *R*_*RSIHR *_usually has less variation in patient allocation, and the values of variation are consistent across all simulation scenarios. Additionally, the patient allocation at *R*_*RSIHR *_is not too extreme. Therefore, *R*_*RSIHR *_provides a good balance between assigning more patients to the better treatment and maintaining the overall power.

**Conclusion:**

The Cook's correction to chi-square test and Williams' correction to log-likelihood-ratio test are generally recommended for hypothesis test in response-adaptive randomization, especially when sample sizes are small. The generalized drop-the-loser urn design is the recommended method for its good overall properties. Also recommended is the use of the *R*_*RSIHR *_allocation target.

## Background

The response-adaptive randomization (RAR) in clinical trials is a class of flexible ways of assigning treatment to new patients sequentially based on available data. The RAR adjusts the allocation probabilities to reflect the interim results of the trial, thereby allowing patients to benefit from the interim knowledge as it accumulates in the trial. In practice, unequal allocation probabilities are generated based on the current assessment of treatment efficacy, which results in more patients being assigned to the treatment that is putatively superior.

Many RAR designs have been proposed over the years [1-13]. The two key issues extensively investigated are the evaluations of parameter estimations and hypothesis testing. Due to the dependency of assigning new patients based on observed data at that time, conventional estimates of treatment effect are often biased; therefore, efforts have been made to quantify and correct estimation bias [14,15]. Recent theoretical works have been focused on solving problems encountered in practice, which includes delayed response, implementation for multi-arm trials, and incorporating covariates, etc. [1,3,11,16-18]. Many recent theoretical developments are summarized in [19]. Additionally, in order to compare treatment efficacies through hypothesis testing, studies have been conducted on power comparisons and sample size calculations under the framework of adaptive randomization [20-24]. However, most of the works are based on large sample sizes, and focus on asymptotic properties [4,12,22,25,26]. But these properties have not been fully studied with small sample sizes. The mathematical challenge imposed by correlated data makes it extremely difficult to derive exact solutions for finite samples. Up to now, only limited results on exact solutions have been available [15,27], and computer simulation has to be relied upon when sample size is small [23,24], which is often the case in early phase II trials.

Each RAR design has its own objective, and there are both advantages and disadvantages associated with that objective. It is not our purpose to give a comprehensive assessment of different designs by comparing their advantages and disadvantages. Instead, the primary objective of the present study is to characterize the small sample properties of RAR based on a frequentist approach. In particular, we focus on comparing the performance of commonly used test statistics in RAR of two-arm comparative trials with a binary outcome. Due to the departure from normality caused by data correlation and the discrete nature of a binary outcome, hypothesis tests usually can not be controlled at any given levels of nominal significance. Thus, to make our simulation comparison more relevant, our assessment of hypothesis testing methods and RAR procedures is based on the calculation of both statistical power and the comparison to the nominal type I error rate. Several RAR methods studied in our simulations can assign patients according to a given allocation target, which may be optimal in terms of maximizing the power or minimizing the expected treatment failure. Therefore, we also compare the properties of test statistics at different optimal allocation targets.

The remaining parts of this paper are organized into 4 sections. In the Methods Section, we introduce the adaptive randomization procedures, the optimal allocation rates, and the test statistics used in the simulation. In the Results Section, we present the simulation results. We provide a discussion and final recommendations regarding the RAR methods and hypothesis tests in the Discussion and Conclusions Sections.

## Methods

In the present section, we briefly describe the randomization methods, asymptotic hypothesis test statistics, and optimal patient allocation targets that are relevant to our simulations. More detailed information can be found in the corresponding references.

### Response-based Adaptive Randomization (RAR)

The RAR procedures investigated in the present study are randomized play-the-winner (RPW) [8,10], drop-the-loser (DL) [28], sequential maximum likelihood estimation (SMLE) [12], doubly-adaptive biased coin [2,3], sequential estimation-adjusted urn (SEU) [13], and generalized drop-the-loser (GDL) [11] designs. RPW, DL, SEU and GDL are all urn models in the sense that treatment assignment for each patient can be obtained by sampling balls from an urn. In the usual clinical trial setting, an urn model consists of one urn with different types of balls that represent the different treatments under study. Patients are assigned to treatments by randomly selecting balls from the urn. Initially, the urn contains an equal number of balls for each of the treatment offered in the trial. With the progress of a clinical trial, certain rules are applied to update the contents of the urn in such a way that favors the selection of balls corresponding to the better treatment. For example, under the RPW design, the observation of a successful treatment response leads to the addition of *a *(>0) balls of the same type to the urn; a lack of success leads to the addition of *b *(>0) balls of the other type to the urn (*a *= *b *= 1 in our simulation). The limiting allocation rate of patients on treatment 1 is *q*_2_/(*q*_1 _+ *q*_2_), where *q*_1 _= 1-*p*_1 _and *q*_2 _= 1-*p*_2 _are failure rates, and *p*_1 _and *p*_2 _are success rates (or response rates) for treatments 1 and 2. In the DL model, patients are assigned to a treatment based on the type of ball that is drawn; however a treatment failure results in the removal of a treatment ball from the urn, and treatment successes are ignored. Due to the finite probabilities of extinction, immigration balls are added to the urn. If an immigration ball is drawn, an additional ball of each type is added. The sampling process is repeated until a treatment ball is drawn. The DL urn design has the same limiting allocation as the RPW urn, but less variability in patient allocation. Both SEU and GDL are urn models allowing fraction number of balls, and can target any allocation rate. For SEU method [13], if the limiting allocation of RPW urn is the target in a two-arm trial, then  balls of type 2 and  balls of type 1 are added to the urn following the allocation of the *i*th patient. Obviously, the response status of the *i*th patient is related to the contents of SEU urn only through the calculation of  and . For a two-arm GDL urn model [11], when a treatment ball is drawn, a new patient is assigned accordingly, but the ball will not be returned to the urn. Depending on the response of the patient, the conditional average numbers of balls being added back to the urn are *b*_1 _and *b*_2 _for treatments 1 and 2, respectively. Therefore, the conditional average numbers of type 1 and type 2 balls being taken out of the urn can be defined as *d*_1 _and *d*_2_, where *d*_1 _= 1-*b*_1 _and *d*_2 _= 1-*b*_2_. Immigration balls are also present in a GDL urn. Whenever an immigration ball is drawn, *a*_1 _and *a*_2 _balls are added for treatments 1 and 2, respectively. Zhang et al [11] have shown that the limiting allocation rate of patients on treatment 1 is(1)

The GDL urn becomes a DL urn when *a*_1 _= 1, *a*_2 _= 1, *b*_1 _= *p*_1_, and *b*_2 _= *p*_2_. Although GDL is a general method with different ways of implementation, a convenient approach is taken in our simulation. When a treatment ball is drawn, the ball is not returned, and no ball is added regardless of the response of the patient. When an immigration ball is drawn, *Cρ*_1 _and *Cρ*_2 _balls of type 1 and 2 are added, where *C *is a constant, and *ρ*_1 _and *ρ*_2 _are allocation targets on treatments 1 and 2, which are estimated sequentially using the maximum likelihood estimates (MLE) [11].

The SMLE and doubly-adaptive biased coin design (DBCD) methods can also target any allocation ratios, and SMLE can be implemented as a special case of DBCD method. In DBCD method, the probability of the (*i*+1)th patient being assigned to treatment 1 is calculated by(2)

where *r*_1 _= *n*_1_*(i)/i *and *ρ*(*i*) are the current allocation rate and estimated allocation rate on treatment 1 [2,3]. The properties of the DBCD depend largely on the selection of *g*, which can be considered as a measuring function for the deviation from the allocation target. In the present study, we use the following function suggested by Hu and Zhang [3]:(3)

where *α *is a tuning parameter. When *α *approaches infinity, the DBCD becomes deterministic and the patients are assigned to the putatively better treatment with probability 1. When *α *equals to 0, the MLE of *ρ *becomes the allocation target, and the DBCD method is essentially the same as the SMLE design proposed by Melfi et al [12].

### Hypothesis Tests for Two-Arm Comparative Trials

In two-arm comparative trials, the results of a binary outcome variable can be summarized in a 2 × 2 contingency table (Table 1). The following hypothesis test is often conducted to compare treatment efficacy:(4)

**Table 1:** Summary of data from a two-arm comparative clinical trial

	Response	Failure	Margins
Treatment 1	*r*_1_	*f*_1_	*n*_1_
Treatment 2	*r*_2_	*f*_2_	*n*-*n*_1 _= *n*_2_

Margins	*r*_1 _+ *r*_2 _= *r*	*n*-*r *= *f*_1 _+ *f*_2 _= *f*	*n*

*n*: total number of patients; *n*_1_, *n*_2_: patients on treatment 1 and 2; *r*: total number of treatment successes; *r*_1_, *r*_2_: number of successes on treatment 1 and 2.

Nine test statistics for the hypothesis test in (4) are given in Table 2. When relative risk (*q*_1_/*q*_2_) and odds ratio (*p*_1_*q*_2_/*q*_1_*p*_2_) are used to quantify the differences between 2 treatment arms, the test statistics are log-relative-risk and log-odds-ratio, *T*_*Risk *_and *T*_*Odds*_, which are asymptotically distributed as chi-square distribution with one degree of freedom (). When simple difference is used to measure the treatment effect, the applicable test statistics are the Wald-type test statistic *T*_*Wald *_and the score-type test statistics *T*_*Chisq*_, where the variance of simple difference in response rates is evaluated at *H*_1 _or *H*_0 _respectively. Additionally, the test statistics based on the logarithm of likelihood ratio (*T*_*LLR*_) can also be constructed. Besides the 5 commonly used test statistics mentioned above, four modified test statistics are also included in Table 2. *T*_*MO *_is a modified log-odds-ratio test proposed by Gart using the approximation of discrete distributions by their continuous analogues [29]. As shown in Table 2, *T*_*MO *_is essentially a modification to *T*_*Odds *_by adding 0.5 to each cell of a 2 × 2 table. Similarly, Agresti and Caffo proposed a modification to *T*_*Wald *_by adding 1 to each cell of a contingency table [30], which results in the test statistic *T*_*MW *_in Table 2. *T*_*MC *_is the Cook's continuity correction to chi-square test statistics *T*_*Chisq*_. Williams provided a modification to log-likelihood-ratio test *T*_*LLR *_[31]. The original test statistic *T*_*LLR *_is improved by multiplying a scale factor such that the null distribution of the new test statistic *T*_*ML *_has the same moments as the chi-square distribution.

**Table 2:** Test statistics

Log-relative-risk	*T*_*Risk*_ = (log(*f*_2_*n*_1_/*f*_1_*n*_2_))^2^/(*r*_1_/*n*_1_*f*_1_ + *r*_2_/*n*_2_*f*_2_)
Log-odds-ratio	*T*_*Odds*_ = (log(*f*_2_* r*_1_*/f*_1_* r*_2_))^2^/(1/*f*_1_ + 1/*f*_2_ + 1/*r*_1_ + 1/r_2_)
Wald-type Z	
Chi-square	*T*_*Chisq*_ = (*n* - 1) (*r*_1_*f*_2_ - *r*_2_*f*_1_)^2^/*rfn*_1_*n*_2_
Log-likelihood-ratio	*T*_*LLR*_ = 2·(*r*_1_ log *r*_1_ + *r*_2_ log *r*_2_ + *f*_1_ log *f*_1_ + *f*_2_ log *f*_2_ - *r* log *r*- *f* log *f* - *n*_1_ log *n*_1_ - *n*_2_ log *n*_2_ + *n* log *n*)
Gart's correction to *T*_*Odds *_[29]	*T*_*MO*_ = (log (*f'*_2_*n'*_1_/*f'*_1_*n'*_2_))^2^/(*r'*_1_/*n'*_1_*f'*_1_ + *r'*_2_/*n'*_2_*f'*_2_)
Agresti's correction to *T*_*Wald*_	
Cook's correction to *T*_*Chisq*_	*T*_*MC*_ = (*n* - 1)(|*r*_1_*f*_2_ - *r*_2_*f*_1_|- 0.5)^2^/*rfn*_1_*n*_2_
William's correction to *T*_*LLR *_[31]	*T*_*ML*_ = [1 + (*n*_2_ - *rf*)(*n*_2_ - *n*_1_*n*_2_)/6*rfn*_1_*n*_2_*n*]^-1^·*T*_*LLR*_

*r'*_1_ = *r*_1_ + 0.5, *r'*_2_ = *r*_2_ + 0.5, *f'*_1_ = *f*_1_ + 0.5, *f'*_2_ = *f*_2_ + 0.5, *r'* = *r* + 1, *f'* + 1, *n'*_1_ = *n*_1_ + 1, *n'*_2_ = *n*_2_ + 1, *n'* = *n* + 2 *r"*_1_ = *r*_1_ + 1, *r"*_2_ = *r*_2_ + 1, *f"*_1_ = *f*_1_ + 1, *f"*_2_ = *f*_2_ + 1, *r"* = *r* + 2, *f"* = *f* + 2, *n"*_1_ = *n*_1_ + 2, *n"*_2_ = *n*_2_ + 2, *n"* = *n* + 4

Since all test statistics in Table 2 are based on , they are asymptotically equivalent and any one of them can be used for large sample sizes. Meanwhile at small sample sizes, an exact test can be conducted if a model is specified for the data given in Table 1. For example, depending on the number of fixed margins predetermined for the design, one of the following three models can be applied [32]:(5)(6)

and(7)

where *h*(*r*_1_|*n*, *n*_1_, *r*) represents the hypergeometric distribution of *r*_1_, *b*(*r*|*n*, *p*) gives the binomial distribution of *r *under the null hypothesis of equal response rates (*H*_0_: *p*_1 _= *p*_2 _= *p*), and *b*(*n*_1_|*n*, *ρ*) denotes the binomial distributions of patients on arm 1 with an allocation ratio of *ρ *(*ρ*_1 _= 0.5 for equal randomization). The p value of exact test can be calculated by maximizing the probability in (5), (6), or (7) over the two nuisance parameters, *p *and *ρ*. However, due to data dependency, none of the above three models are directly applicable in adaptive randomization. For example, the allocation ratio *ρ *in adaptive randomization is a random variable with unknown distribution, and the binomial distribution of *n*_1 _assumed in model (7) is not valid even when the null hypothesis is true. Therefore, in adaptive randomization, unconditional exact tests are not available and asymptotic test statistics such as the ones in Table 2 are required for testing the hypothesis in (4).

### Optimal Allocation Ratios

The SMLE, DBCD, SEU, and GDL methods can be utilized to allocate patients based on different allocation targets. The allocation targets simulated in the present study are summarized in Table 3, where *R*_*Risk*_, *R*_*Odds*_, *R*_*Wald*_, *R*_*Chisq*_, and *R*_*LLR *_are optimal allocation ratios maximizing the power of *T*_*Risk*_, *T*_*Odds*_, *T*_*Wald*_, *T*_*Chisq*_, and *T*_*LLR *_respectively, at fixed sample size. The derivation of *T*_*Risk*_, *T*_*Odds*_, *T*_*Wald*_, *T*_*Chisq*_, and *T*_*LLR *_can be found in [33,34], which is equivalent to minimizing the variance of corresponding test statistic at a fixed total sample size, and consequently the power of that test statistic is maximized. *R*_*RSIHR *_is a recently proposed allocation target that minimizes the expected total number of failures among all trials with the same power [15,33]. The general theoretical framework and the practical implementation of optimal allocation in *k*-arm trials with binary outcomes are discussed and demonstrated by Tymofyeyev et al [35], where the optimization can be conducted over different goals. In practice, the performance of the methodology depends on the chosen RAR procedure. The present simulation study only focuses on two-arm trials, with a goal of maximizing the power or minimizing the total number of failures.

**Table 3:** Allocation targets

Optimal allocation ratio (*n*_1_/*n*_2_) for maximizing powers	
R_*Risk*_	
*R*_*Odds *_/R_*Chisq*_	
*R*_*Wald *_/R_*Neyman*_	
R_*LLR*_	{*q*_2_ - *p*_2_ exp[*I*_1_ - *I*_2_/(*p*_2_ - *p*_1_)]}/{-*q*_1_ + *p*_1_ exp[*I*_1_ - *I*_2_/(*p*_2_ - *p*_1_)]}

Other allocation targets	

*R*_*RPW*_/R_*DL*_	*q*_2_/*q*_1_
R_*RSIHR*_	(Minimize the number of failure at fixed power of *T*_*Wald*_)

*I*_1_ = *p*_1_ log(*p*_1_) + *q*_1_ log (*q*_1_), *I*_2_ = *p*_2_ log(*p*_2_) + *q*_2_ log(*q*_2_)

## Results

Simulations are conducted at different total numbers of patients ranging from 20 to 200. To simplify the presentation, the results for trials with 30 patients are shown here. When patients are less than 30, adaptive randomization is generally not recommended. For sample size of 100 or larger, all methods yield similar properties in general. For all of the urn models, one ball for each treatment is consistently used as the initial contents of the urn. The number of immigration balls is 1 for both the DL and GDL urns. The tuning parameter of DBCD, *α*, is fixed at 0 or 2. When *α *is 0, it results in the SMLE method. The value of the constant *C *in GDL is 2, which is equivalent to adding 2 treatment balls on average when an immigration ball is drawn. All simulation results are calculated based on 10,000 replicates.

For the purpose of comparison, the true allocation rates are shown in Table 4, and the simulated results for allocation rates on arm 1 are shown in Table 5. Among all RAR methods, DBCD has the best ability to attain the true allocation target. The comparison between SMLE and DBCD shows that, the allocation becomes more unbalanced and the variation of DBCD decreases with increasing value of tuning exponent *α*. On the other hand, the patient allocation of SEU results in more balanced mean allocation between two arms with a much larger variation as compared with other RAR methods. The GDL has the lowest variation among the four sequential RAR methods. When *R*_*RPW *_(the same as *R*_*DL*_) is the allocation target, DL urn method has the lowest variation in patient allocation, which is consistent with the fact that the lower bound of the estimate of Var(*R*_*RPW*_) is attained by DL urn [4]. The comparison among allocation targets shows that *R*_*LLR *_has the lowest variation in patient allocation, and the highest variation is usually found at *R*_*RPW *_or *R*_*Risk*_. However, *R*_*RPW *_and *R*_*Risk *_are usually the top two allocation targets that assign more patients to the better treatment. *R*_*Wald*_, *R*_*Odds*_, and *RLLR *assigns more patients to the worse arm in some simulation cases. Among the three allocation targets that assign more patients to the better treatment (*R*_*RSIHR*_, *R*_*Risk *_and *R*_*RPW*_), *R*_*RSIHR *_has a stable and often the lowest variation in patient allocation.

**Table 4:** Asymptotic allocation rates on arm 1 calculated from true *p*_1_**and *p***_2_

*p*_1_	0.100	0.100	0.100	0.100	0.300	0.300	0.300	0.500	0.500	0.700
*p*_2_	0.300	0.500	0.700	0.900	0.500	0.700	0.900	0.700	0.900	0.900
*R*_*Wald *_/*R*_*Neyman*_	0.396	0.375	0.396	0.500	0.478	0.500	0.604	0.522	0.625	0.604
R_*Risk*_	0.337	0.250	0.179	0.100	0.396	0.300	0.179	0.396	0.250	0.337
*R*_*Odds *_/*R*_*Chisq*_	0.604	0.625	0.604	0.500	0.522	0.500	0.396	0.478	0.375	0.396
R_*LLR*_	0.534	0.538	0.528	0.500	0.507	0.500	0.472	0.493	0.462	0.466
R_*RSIHR*_	0.366	0.309	0.274	0.250	0.436	0.396	0.366	0.458	0.427	0.469
*R*_*RPW *_/*R*_*DL*_	0.438	0.357	0.250	0.100	0.417	0.300	0.125	0.375	0.167	0.250

**Table 5:** Mean and standard deviation (in parenthesis) of allocation rate on arm 1 for *n *= 30.

Null	*p*_1_	0.2	0.3	0.5	0.7	0.8
	*p*_2_	0.2	0.3	0.5	0.7	0.8
Urn	*RPW*	0.500(0.081)	0.500(0.095)	0.500(0.129)	0.500(0.179)	0.500(0.209)
	*DL*	0.500(0.048)	0.500(0.058)	0.500(0.078)	0.500(0.092)	0.500(0.097)

SMLE	*R*_*Wald*_	0.500(0.106)	0.500(0.103)	0.500(0.098)	0.500(0.103)	0.500(0.106)
	*R*_*Risk*_	0.500(0.130)	0.500(0.134)	0.500(0.140)	0.500(0.151)	0.500(0.158)
	*R*_*Odds*_	0.500(0.109)	0.500(0.098)	0.500(0.091)	0.500(0.099)	0.500(0.109)
	*R*_*LLR*_	0.500(0.093)	0.500(0.092)	0.500(0.091)	0.500(0.093)	0.500(0.094)
	*R*_*RSIHR*_	0.500(0.117)	0.500(0.116)	0.500(0.109)	0.500(0.106)	0.500(0.102)
	*R*_*RPW*_	0.500(0.100)	0.500(0.109)	0.500(0.131)	0.500(0.166)	0.500(0.192)

DBCD	*R*_*Wald*_	0.500(0.090)	0.500(0.075)	0.500(0.055)	0.500(0.075)	0.500(0.090)
	*R*_*Risk*_	0.500(0.126)	0.500(0.124)	0.500(0.123)	0.500(0.127)	0.500(0.140)
	*R*_*Odds*_	0.500(0.082)	0.500(0.061)	0.500(0.047)	0.500(0.061)	0.500(0.082)
	*R*_*LLR*_	0.500(0.049)	0.500(0.046)	0.500(0.044)	0.500(0.047)	0.500(0.049)
	*R*_*RSIHR*_	0.500(0.107)	0.500(0.099)	0.500(0.078)	0.500(0.060)	0.500(0.054)
	*R*_*RPW*_	0.500(0.064)	0.500(0.074)	0.500(0.104)	0.500(0.148)	0.500(0.185)

SEU	*R*_*Wald*_	0.500(0.113)	0.500(0.106)	0.500(0.098)	0.500(0.106)	0.500(0.114)
	*R*_*Risk*_	0.500(0.155)	0.500(0.168)	0.500(0.195)	0.500(0.223)	0.500(0.237)
	*R*_*Odds*_	0.500(0.101)	0.500(0.104)	0.500(0.130)	0.500(0.176)	0.500(0.196)
	*R*_*LLR*_	0.500(0.093)	0.500(0.091)	0.500(0.091)	0.500(0.093)	0.500(0.092)
	*R*_*RSIHR*_	0.500(0.149)	0.500(0.146)	0.500(0.131)	0.500(0.116)	0.500(0.106)
	*R*_*RPW*_	0.500(0.135)	0.500(0.155)	0.500(0.192)	0.500(0.222)	0.500(0.233)

GDL	*R*_*Wald*_	0.500(0.056)	0.500(0.046)	0.500(0.033)	0.500(0.047)	0.500(0.056)
	*R*_*Risk*_	0.500(0.106)	0.500(0.114)	0.500(0.128)	0.500(0.144)	0.500(0.154)
	*R*_*Odds*_	0.500(0.040)	0.500(0.035)	0.500(0.055)	0.500(0.090)	0.500(0.112)
	*R*_*LLR*_	0.500(0.029)	0.500(0.026)	0.500(0.024)	0.500(0.026)	0.500(0.029)
	*R*_*RSIHR*_	0.500(0.073)	0.500(0.070)	0.500(0.058)	0.500(0.045)	0.500(0.039)
	*R*_*RPW*_	0.500(0.053)	0.500(0.065)	0.500(0.088)	0.500(0.116)	0.500(0.133)

Alternative	*p*_1_	0.1	0.1	0.1	0.1	0.3
	*p*_2_	0.3	0.5	0.7	0.9	0.5

Urn	*RPW*	0.444(0.080)	0.375(0.092)	0.287(0.096)	0.181(0.088)	0.430(0.109)
	*DL*	0.447(0.046)	0.383(0.055)	0.316(0.056)	0.249(0.053)	0.437(0.067)

SMLE	*R*_*Wald*_	0.440(0.100)	0.424(0.098)	0.441(0.100)	0.501(0.102)	0.483(0.101)
	*R*_*Risk*_	0.397(0.117)	0.325(0.107)	0.259(0.095)	0.186(0.079)	0.415(0.133)
	*R*_*Odds*_	0.562(0.110)	0.577(0.107)	0.561(0.110)	0.499(0.126)	0.517(0.095)
	*R*_*LLR*_	0.519(0.094)	0.522(0.094)	0.515(0.094)	0.499(0.095)	0.506(0.092)
	*R*_*RSIHR*_	0.417(0.108)	0.369(0.100)	0.335(0.093)	0.312(0.087)	0.447(0.112)
	*R*_*RPW*_	0.447(0.099)	0.384(0.105)	0.297(0.106)	0.179(0.091)	0.434(0.117)

DBCD	*R*_*Wald*_	0.417(0.081)	0.393(0.073)	0.416(0.081)	0.499(0.095)	0.475(0.065)
	*R*_*Risk*_	0.371(0.106)	0.285(0.086)	0.216(0.071)	0.138(0.054)	0.394(0.116)
	*R*_*Odds*_	0.585(0.085)	0.607(0.078)	0.586(0.086)	0.499(0.110)	0.520(0.053)
	*R*_*LLR*_	0.474(0.048)	0.468(0.046)	0.477(0.047)	0.500(0.047)	0.493(0.045)
	*R*_*RSIHR*_	0.392(0.093)	0.332(0.077)	0.297(0.069)	0.273(0.063)	0.431(0.088)
	*R*_*RPW*_	0.440(0.063)	0.366(0.072)	0.266(0.078)	0.129(0.064)	0.422(0.087)

SEU	*R*_*Wald*_	0.476(0.113)	0.464(0.110)	0.473(0.113)	0.505(0.117)	0.493(0.104)
	*R*_*Risk*_	0.433(0.143)	0.361(0.130)	0.296(0.115)	0.234(0.091)	0.440(0.166)
	*R*_*Odds*_	0.514(0.108)	0.497(0.124)	0.462(0.143)	0.388(0.137)	0.489(0.119)
	*R*_*LLR*_	0.510(0.093)	0.512(0.094)	0.508(0.093)	0.501(0.094)	0.503(0.092)
	*R*_*RSIHR*_	0.461(0.143)	0.425(0.130)	0.402(0.122)	0.383(0.113)	0.475(0.136)
	*R*_*RPW*_	0.469(0.129)	0.424(0.136)	0.367(0.135)	0.294(0.113)	0.462(0.164)

GDL	*R*_*Wald*_	0.450(0.051)	0.437(0.046)	0.452(0.051)	0.500(0.058)	0.486(0.040)
	*R*_*Risk*_	0.397(0.093)	0.320(0.085)	0.251(0.071)	0.181(0.055)	0.407(0.114)
	*R*_*Odds*_	0.527(0.043)	0.508(0.053)	0.454(0.072)	0.341(0.080)	0.484(0.045)
	*R*_*LLR*_	0.517(0.027)	0.521(0.026)	0.515(0.027)	0.500(0.028)	0.505(0.024)
	*R*_*RSIHR*_	0.431(0.065)	0.389(0.057)	0.362(0.051)	0.342(0.047)	0.454(0.062)
	*R*_*RPW*_	0.454(0.052)	0.399(0.063)	0.329(0.067)	0.236(0.059)	0.444(0.075)

Alternative	*p*_1_	0.3	0.3	0.5	0.5	0.7
	*p*_2_	0.7	0.9	0.7	0.9	0.9

Urn	*RPW*	0.341(0.120)	0.227(0.123)	0.411(0.147)	0.288(0.160)	0.375(0.202)
	*DL*	0.363(0.071)	0.290(0.066)	0.424(0.082)	0.343(0.082)	0.416(0.092)

SMLE	*R*_*Wald*_	0.500(0.104)	0.559(0.100)	0.517(0.100)	0.576(0.099)	0.558(0.101)
	*R*_*Risk*_	0.334(0.124)	0.238(0.109)	0.411(0.139)	0.298(0.131)	0.375(0.149)
	*R*_*Odds*_	0.500(0.098)	0.438(0.109)	0.485(0.095)	0.423(0.107)	0.438(0.109)
	*R*_*LLR*_	0.499(0.091)	0.483(0.093)	0.495(0.092)	0.477(0.094)	0.481(0.094)
	*R*_*RSIHR*_	0.408(0.107)	0.378(0.103)	0.459(0.106)	0.429(0.105)	0.468(0.101)
	*R*_*RPW*_	0.343(0.122)	0.209(0.110)	0.405(0.141)	0.255(0.136)	0.332(0.174)

DBCD	*R*_*Wald*_	0.500(0.075)	0.585(0.081)	0.525(0.065)	0.607(0.073)	0.584(0.081)
	*R*_*Risk*_	0.300(0.104)	0.187(0.083)	0.391(0.118)	0.250(0.108)	0.337(0.130)
	*R*_*Odds*_	0.501(0.061)	0.413(0.086)	0.480(0.054)	0.394(0.079)	0.414(0.084)
	*R*_*LLR*_	0.500(0.046)	0.524(0.047)	0.508(0.045)	0.532(0.046)	0.527(0.048)
	*R*_*RSIHR*_	0.387(0.080)	0.353(0.075)	0.453(0.069)	0.417(0.066)	0.464(0.055)
	*R*_*RPW*_	0.317(0.095)	0.157(0.082)	0.386(0.118)	0.201(0.112)	0.284(0.158)

SEU	*R*_*Wald*_	0.502(0.106)	0.535(0.108)	0.509(0.102)	0.540(0.102)	0.532(0.108)
	*R*_*Risk*_	0.365(0.154)	0.280(0.126)	0.437(0.197)	0.337(0.171)	0.411(0.212)
	*R*_*Odds*_	0.453(0.134)	0.384(0.131)	0.469(0.150)	0.399(0.146)	0.438(0.177)
	*R*_*LLR*_	0.500(0.091)	0.493(0.094)	0.498(0.093)	0.490(0.094)	0.490(0.092)
	*R*_*RSIHR*_	0.449(0.126)	0.429(0.121)	0.479(0.124)	0.460(0.117)	0.481(0.109)
	*R*_*RPW*_	0.408(0.162)	0.326(0.141)	0.456(0.197)	0.366(0.173)	0.423(0.208)

GDL	*R*_*Wald*_	0.499(0.047)	0.548(0.052)	0.514(0.041)	0.562(0.046)	0.548(0.051)
	*R*_*Risk*_	0.319(0.104)	0.220(0.078)	0.397(0.128)	0.274(0.104)	0.356(0.138)
	*R*_*Odds*_	0.431(0.064)	0.327(0.072)	0.447(0.071)	0.342(0.080)	0.390(0.102)
	*R*_*LLR*_	0.500(0.026)	0.485(0.027)	0.495(0.025)	0.479(0.026)	0.483(0.028)
	*R*_*RSIHR*_	0.423(0.056)	0.398(0.052)	0.466(0.052)	0.440(0.046)	0.472(0.038)
	*R*_*RPW*_	0.367(0.082)	0.263(0.073)	0.420(0.098)	0.303(0.092)	0.370(0.121)

The simulation results are obtained for five null cases and ten alternative cases, and Table 6 gives the summary by averaging the results over the five null cases and the ten alternative cases for a given RAR method and at a given allocation target. Detailed simulation results for each test statistic are shown in Tables 7, 8, 9, 10, 11, 12 with one table for each of the six allocation targets. To simplify the presentation, the results are shown only for the four modified test statistics *T*_*MW*_, *T*_*MO*_, *T*_*MC*_, *T*_*ML*_, and the log-relative-risk test statistic *T*_*Risk *_because they tend to have better performance than the four corresponding unmodified tests. The qualitative comparisons among test statistics, RAR methods, and allocation targets can be made based on the results in Table 6.

**Table 6:** The mean and standard deviation (in parenthesis) of type I error and power.

		Type I error of test statistics					
**Target**	***Method***	**T**_*MW*_	**T**_*RISK*_	**T**_*MO*_	**T**_*MC*_	**T**_*ML*_	**Row Mean**

	*SMLE*	4.4(1.1)	4.6(4.1)	2.0(1.4)	5.0(0.6)	6.8(0.9)	4.6(2.4)
	*DBCD*	4.3(1.4)	5.1(5.1)	1.7(1.7)	4.8(1.2)	7.2(0.8)	4.6(2.9)
*R*_*Wald*_	*SEU*	4.0(0.9)	3.4(2.4)	2.3(1.2)	4.8(0.2)	5.6(0.6)	4.0(1.7)
	*GDL*	4.4(0.8)	3.7(3.1)	2.1(1.6)	5.2(0.4)	6.6(1.0)	4.4(2.2)
	
	*Mean*	4.3(1.0)	4.2(3.6)	2.0(1.4)	5.0(0.7)	6.5(1.0)	4.4(2.3)

	*SMLE*	4.4(1.4)	8.6(3.5)	2.4(1.8)	5.5(1.4)	6.0(1.0)	5.4(2.8)
	*DBCD*	4.6(2.0)	10.2(4.4)	2.6(2.3)	5.7(2.2)	6.5(1.4)	5.9(3.5)
*R*_*Risk*_	*SEU*	3.7(0.8)	7.6(2.3)	2.1(0.8)	5.4(1.3)	5.1(0.4)	4.8(2.2)
	*GDL*	4.2(1.3)	7.9(2.4)	2.4(1.9)	5.4(1.6)	5.8(1.4)	5.1(2.5)
	
	*Mean*	4.2(1.3)	8.6(3.1)	2.4(1.7)	5.5(1.5)	5.9(1.2)	5.3(2.8)

	*SMLE*	3.7(0.6)	2.4(0.5)	2.9(0.5)	4.8(0.4)	4.5(0.4)	3.7(1.0)
	*DBCD*	3.6(0.7)	2.1(0.8)	3.1(0.7)	4.7(0.3)	4.1(0.2)	3.5(1.1)
*R*_*Odds*_	*SEU*	3.6(0.5)	3.6(0.8)	2.3(0.7)	4.7(0.3)	4.9(0.7)	3.8(1.1)
	*GDL*	3.7(0.8)	3.4(0.8)	3.0(1.1)	5.1(0.4)	4.5(0.4)	3.9(1.0)
	
	*Mean*	3.7(0.6)	2.9(0.9)	2.8(0.8)	4.9(0.4)	4.5(0.5)	3.7(1.1)

	*SMLE*	4.0(0.6)	2.7(1.2)	2.7(1.0)	5.0(0.2)	5.2(0.6)	3.9(1.3)
	*DBCD*	4.2(0.8)	3.3(2.6)	2.4(1.5)	5.0(0.4)	6.1(0.8)	4.2(1.9)
*R*_*LLR*_	*SEU*	4.0(0.6)	2.8(1.6)	2.4(1.0)	4.9(0.2)	5.4(0.8)	3.9(1.5)
	*GDL*	3.7(0.5)	2.5(1.3)	2.7(1.2)	4.9(0.4)	5.4(0.9)	3.8(1.5)
	
	*Mean*	3.9(0.6)	2.8(1.6)	2.5(1.1)	5.0(0.3)	5.6(0.8)	4.0(1.5)

	*SMLE*	4.2(1.1)	6.2(4.0)	2.3(1.5)	5.2(0.8)	6.1(0.7)	4.8(2.4)
	*DBCD*	4.3(1.5)	6.9(5.2)	2.0(1.6)	5.2(1.3)	6.5(1.1)	5.0(3.0)
*R*_*RSIHR*_	*SEU*	3.9(0.8)	4.8(3.4)	2.3(1.0)	4.8(0.4)	5.5(0.5)	4.3(1.9)
	*GDL*	4.3(0.9)	4.7(3.0)	2.2(1.6)	5.1(0.6)	6.1(0.9)	4.5(2.0)
	
	*Mean*	4.2(1.0)	5.7(3.8)	2.2(1.3)	5.1(0.8)	6.1(0.8)	4.6(2.3)

	*RPW*	4.2(0.8)	6.2(0.5)	2.5(1.6)	5.5(1.4)	5.4(0.8)	4.8(1.7)
	*DL*	4.3(0.8)	4.8(1.0)	2.6(1.7)	5.3(0.9)	5.3(0.4)	4.5(1.4)
	*SMLE*	4.2(0.9)	6.5(0.6)	2.8(1.8)	5.4(1.6)	5.1(0.8)	4.8(1.7)
*R*_*RPW*_	*DBCD*	4.3(0.9)	6.7(1.0)	2.9(2.1)	5.7(1.8)	4.8(1.0)	4.9(1.9)
	*SEU*	3.8(0.6)	5.7(1.3)	2.2(0.6)	5.4(0.8)	5.1(0.6)	4.5(1.5)
	*GDL*	4.0(0.8)	5.1(0.6)	2.7(1.6)	5.2(0.7)	5.0(0.8)	4.4(1.3)
	
	*Mean*	4.1(0.8)	5.8(1.1)	2.6(1.5)	5.4(1.2)	5.1(0.7)	4.6(1.6)

Equal Allocation		4.0(0.5)	2.9(1.7)	2.4(1.0)	5.0(0.2)	5.6(0.8)	4.0(1.5)

		Power of test statistics					

**Target**	***Method***	**T**_*MW*_	**T**_*RISK*_	**T**_*MO*_	**T**_*MC*_	**T**_*ML*_	**Row Mean**

	*SMLE*	56.6(34.1)	48.6(35.2)	48.5(36.8)	57.6(33.4)	59.4(31.9)	54.2(33.2)
	*DBCD*	56.9(34.4)	49.5(35.9)	48.0(37.6)	57.7(33.9)	60.2(31.8)	54.5(33.7)
*R*_*Wald*_	*SEU*	56.0(34.0)	47.7(34.8)	49.6(36.1)	57.5(33.0)	58.4(32.3)	53.8(32.9)
	*GDL*	57.3(34.0)	50.0(36.2)	50.6(36.9)	58.4(33.2)	60.0(32.0)	55.3(33.3)
	
	*Mean*	56.7(32.8)	49.0(34.2)	49.2(35.4)	57.8(32.1)	59.5(30.7)	54.4(33.0)

	*SMLE*	53.4(33.2)	57.9(31.5)	45.4(35.2)	56.2(32.7)	55.1(31.1)	53.6(31.7)
	*DBCD*	53.3(33.4)	60.0(30.5)	43.7(36.0)	56.5(32.9)	55.0(31.1)	53.7(31.9)
*R*_*Risk*_	*SEU*	52.5(32.8)	55.3(32.2)	45.9(34.1)	55.2(32.1)	54.2(31.2)	52.6(31.3)
	*GDL*	53.2(33.3)	58.1(31.6)	45.8(35.8)	56.5(32.6)	55.2(31.7)	53.8(31.9)
	
	*Mean*	53.1(31.9)	57.8(30.3)	45.2(33.9)	56.1(31.3)	54.9(30.1)	53.4(31.5)

	*SMLE*	54.6(33.9)	47.1(34.3)	52.1(34.9)	57.6(32.6)	56.4(32.9)	53.6(32.5)
	*DBCD*	54.8(34.2)	47.3(35.2)	53.4(34.5)	57.8(32.7)	56.5(33.4)	53.9(32.8)
*R*_*Odds*_	*SEU*	54.8(33.5)	50.8(33.8)	50.4(34.8)	57.5(32.5)	56.6(32.2)	54.0(32.1)
	*GDL*	54.6(34.2)	53.0(34.6)	52.5(35.0)	58.1(32.7)	56.8(33.0)	55.0(32.5)
	
	*Mean*	54.7(32.6)	49.5(33.2)	52.1(33.4)	57.8(31.4)	56.6(31.6)	54.1(32.3)

	*SMLE*	55.9(33.9)	48.4(35.0)	51.6(35.6)	58.0(32.8)	58.0(32.6)	54.4(32.8)
	*DBCD*	57.2(34.0)	49.9(35.9)	51.4(36.6)	58.6(33.1)	60.0(32.2)	55.4(33.2)
*R*_*LLR*_	*SEU*	56.1(33.9)	48.5(34.8)	51.2(35.7)	58.1(32.8)	58.2(32.5)	54.4(32.8)
	*GDL*	56.4(34.1)	50.4(35.8)	53.1(35.9)	58.9(33.1)	59.5(32.5)	55.7(33.1)
	
	Mean	56.4(32.6)	49.3(34.0)	51.8(34.6)	58.4(31.7)	58.9(31.2)	55.0(32.7)

	*SMLE*	56.0(33.9)	54.8(33.7)	48.7(36.4)	57.5(33.2)	58.4(32.0)	55.1(32.6)
	*DBCD*	56.8(34.0)	56.3(33.4)	48.2(37.0)	58.2(33.2)	59.4(31.8)	55.7(32.8)
*R*_*RSIHR*_	*SEU*	54.5(33.8)	50.5(34.5)	48.6(35.8)	56.4(33.0)	56.6(32.4)	53.3(32.7)
	*GDL*	57.4(33.7)	54.4(34.5)	50.6(36.6)	58.7(33.0)	59.7(32.1)	56.2(32.8)
	
	Mean	56.2(32.6)	54.0(32.8)	49.0(35.0)	57.7(31.8)	58.5(30.8)	55.1(32.5)

	*RPW*	52.4(32.3)	55.9(32.1)	46.3(34.1)	55.8(32.1)	52.9(30.1)	52.7(31.0)
	*DL*	56.0(33.5)	55.9(33.4)	50.0(36.1)	58.2(32.6)	57.4(32.5)	55.5(32.4)
	*SMLE*	51.7(32.3)	56.2(31.8)	46.7(33.7)	55.7(31.9)	51.7(30.2)	52.4(30.9)
*R*_*RPW*_	*DBCD*	51.2(31.8)	57.3(31.2)	47.0(34.1)	56.0(31.5)	48.3(29.2)	52.0(30.6)
	*SEU*	54.0(33.1)	54.0(32.7)	48.3(34.4)	56.7(32.1)	55.9(31.7)	53.8(31.6)
	*GDL*	54.6(33.5)	56.0(33.0)	50.2(35.3)	57.8(32.4)	56.4(32.3)	55.0(32.0)
	
	Mean	53.3(31.4)	55.9(31.0)	48.1(33.2)	56.7(30.7)	53.8(29.8)	53.5(31.2)

Equal Allocation		56.2(33.9)	48.5(35.0)	50.9(35.9)	58.1(32.9)	58.4(32.4)	54.4(32.9)

Mean values are calculated by averaging simulation results over the five null cases and the ten alternative cases of simulation scenarios listed in Tables 7-12. All results have been multiplied by 100% (alpha = 0.05, *n *= 30).

**Table 7:** Power and type I error at *R*_*Wald *_(alpha = 0.05, *n *= 30).

*p*_1_		0.200	0.300	0.500	0.700	0.800	0.100	0.100	0.100	0.100	0.300	0.300	0.300	0.500	0.500	0.700
*p*_2_		0.200	0.300	0.500	0.700	0.800	0.300	0.500	0.700	0.900	0.500	0.700	0.900	0.700	0.900	0.900
	*T*_*MW*_	0.031	0.048	0.056	0.050	0.033	0.196	0.674	0.953	0.999	0.201	0.600	0.950	0.203	0.680	0.202
	*T*_*Risk*_	0.102	0.072	0.039	0.014	0.003	0.326	0.693	0.940	0.996	0.181	0.501	0.798	0.113	0.288	0.024
SMLE	*T*_*MO*_	0.007	0.022	0.041	0.024	0.007	0.063	0.492	0.928	0.999	0.162	0.563	0.923	0.161	0.495	0.069
	*T*_*MC*_	0.044	0.052	0.056	0.055	0.044	0.231	0.689	0.954	0.999	0.203	0.601	0.952	0.205	0.693	0.235
	*T*_*ML*_	0.074	0.066	0.055	0.067	0.079	0.308	0.709	0.954	0.999	0.203	0.595	0.951	0.205	0.711	0.309

	*T*_*MW*_	0.029	0.050	0.057	0.052	0.026	0.186	0.685	0.957	0.999	0.212	0.607	0.958	0.206	0.696	0.191
	*T*_*Risk*_	0.120	0.085	0.041	0.008	0.001	0.361	0.721	0.954	0.998	0.204	0.524	0.811	0.109	0.257	0.010
DBCD	*T*_*MO*_	0.004	0.017	0.045	0.017	0.003	0.041	0.462	0.933	0.999	0.169	0.587	0.934	0.164	0.475	0.042
	*T*_*MC*_	0.037	0.056	0.058	0.056	0.034	0.211	0.696	0.958	0.999	0.215	0.607	0.959	0.208	0.706	0.215
	*T*_*ML*_	0.077	0.074	0.059	0.073	0.077	0.311	0.718	0.958	0.999	0.217	0.607	0.959	0.210	0.727	0.315

	*T*_*MW*_	0.031	0.045	0.048	0.044	0.030	0.200	0.655	0.946	0.999	0.190	0.583	0.948	0.191	0.675	0.213
	*T*_*Risk*_	0.067	0.048	0.033	0.016	0.006	0.259	0.646	0.922	0.991	0.154	0.486	0.812	0.114	0.342	0.046
SEU	*T*_*MO*_	0.013	0.026	0.039	0.027	0.011	0.094	0.522	0.921	0.999	0.158	0.553	0.926	0.157	0.533	0.095
	*T*_*MC*_	0.046	0.051	0.049	0.050	0.046	0.248	0.675	0.949	0.999	0.195	0.585	0.950	0.195	0.698	0.258
	*T*_*ML*_	0.062	0.055	0.047	0.055	0.062	0.285	0.683	0.947	0.999	0.190	0.577	0.949	0.193	0.710	0.305

	*T*_*MW*_	0.036	0.051	0.051	0.049	0.034	0.223	0.696	0.954	1.000	0.195	0.601	0.958	0.200	0.692	0.214
	*T*_*Risk*_	0.075	0.060	0.040	0.010	0.001	0.309	0.703	0.949	0.999	0.184	0.543	0.868	0.124	0.304	0.015
GDL	*T*_*MO*_	0.007	0.022	0.046	0.023	0.006	0.077	0.549	0.937	0.999	0.167	0.588	0.945	0.169	0.547	0.077
	*T*_*MC*_	0.048	0.057	0.051	0.055	0.047	0.260	0.708	0.955	1.000	0.198	0.602	0.960	0.204	0.705	0.253
	*TML*	0.074	0.064	0.052	0.063	0.076	0.319	0.721	0.956	1.000	0.200	0.602	0.960	0.205	0.720	0.314

For each RAR methods, the results of the following 5 test statistics are shown: Agresti's correction to Wald-type Z test *T*_*MW*_, log-relative-risk test *T*_*Risk*_, Gart's correction to log-odds-ratio test *T*_*MO*_, Cook's correction to chi-square test *T*_*MC*_, and Williams' correction log-likelihood-ratio test *T*_*ML*_.

**Table 8:** Power and type I error at *R*_*Risk *_(alpha = 0.05, *n *= 30).

*p*_1_		0.200	0.300	0.500	0.700	0.800	0.100	0.100	0.100	0.100	0.300	0.300	0.300	0.500	0.500	0.700
*p*_2_		0.200	0.300	0.500	0.700	0.800	0.300	0.500	0.700	0.900	0.500	0.700	0.900	0.700	0.900	0.900
	*T*_*MW*_	0.024	0.045	0.061	0.051	0.041	0.156	0.615	0.923	0.990	0.185	0.560	0.898	0.189	0.611	0.214
	*T*_*Risk*_	0.136	0.105	0.078	0.061	0.050	0.363	0.716	0.945	0.997	0.230	0.588	0.923	0.206	0.612	0.210
SMLE	*T*_*MO*_	0.002	0.008	0.032	0.039	0.040	0.022	0.278	0.792	0.988	0.096	0.466	0.903	0.157	0.615	0.220
	*T*_*MC*_	0.033	0.047	0.060	0.064	0.068	0.177	0.615	0.923	0.996	0.183	0.570	0.939	0.202	0.701	0.316
	*T*_*ML*_	0.069	0.071	0.061	0.049	0.051	0.278	0.659	0.921	0.975	0.195	0.543	0.883	0.179	0.621	0.253

	*T*_*MW*_	0.018	0.046	0.072	0.054	0.042	0.134	0.617	0.931	0.993	0.198	0.565	0.896	0.199	0.586	0.207
	*T*_*Risk*_	0.166	0.123	0.091	0.066	0.062	0.402	0.744	0.951	0.998	0.253	0.606	0.926	0.225	0.649	0.243
DBCD	*T*_*MO*_	0.001	0.003	0.030	0.046	0.049	0.004	0.164	0.746	0.994	0.074	0.457	0.904	0.158	0.623	0.248
	*T*_*MC*_	0.023	0.047	0.070	0.068	0.077	0.148	0.612	0.928	0.998	0.193	0.575	0.940	0.218	0.707	0.327
	*T*_*ML*_	0.071	0.083	0.071	0.050	0.050	0.278	0.665	0.928	0.979	0.207	0.549	0.880	0.184	0.596	0.240

	*T*_*MW*_	0.026	0.039	0.045	0.043	0.032	0.172	0.598	0.903	0.988	0.178	0.537	0.888	0.183	0.606	0.198
	*T*_*Risk*_	0.105	0.092	0.075	0.059	0.049	0.307	0.686	0.935	0.996	0.201	0.546	0.903	0.186	0.581	0.193
SEU	*T*_*MO*_	0.009	0.018	0.029	0.027	0.023	0.062	0.372	0.794	0.986	0.121	0.468	0.887	0.146	0.582	0.176
	*T*_*MC*_	0.041	0.044	0.050	0.064	0.070	0.209	0.605	0.903	0.994	0.178	0.542	0.922	0.194	0.681	0.289
	*T*_*ML*_	0.057	0.052	0.047	0.049	0.048	0.266	0.640	0.900	0.981	0.183	0.526	0.879	0.178	0.624	0.245

	*T*_*MW*_	0.023	0.043	0.059	0.047	0.038	0.168	0.617	0.929	0.993	0.182	0.558	0.902	0.196	0.580	0.195
	*T*_*Risk*_	0.113	0.092	0.076	0.062	0.053	0.347	0.720	0.950	0.998	0.227	0.593	0.928	0.220	0.617	0.213
GDL	*T*_*MO*_	0.001	0.006	0.031	0.040	0.042	0.016	0.283	0.831	0.994	0.094	0.473	0.908	0.161	0.604	0.220
	*T*_*MC*_	0.030	0.047	0.058	0.064	0.070	0.194	0.618	0.928	0.998	0.180	0.567	0.943	0.214	0.696	0.311
	*T*_*ML*_	0.077	0.068	0.058	0.044	0.045	0.292	0.653	0.927	0.990	0.189	0.540	0.901	0.182	0.606	0.236

**Table 9:** Power and type I error at *R*_*Odds *_(alpha = 0.05, *n *= 30).

*p*_1_		0.200	0.300	0.500	0.700	0.800	0.100	0.100	0.100	0.100	0.300	0.300	0.300	0.500	0.500	0.700
*p*_2_		0.200	0.300	0.500	0.700	0.800	0.300	0.500	0.700	0.900	0.500	0.700	0.900	0.700	0.900	0.900
	*T*_*MW*_	0.030	0.040	0.042	0.040	0.031	0.202	0.630	0.935	0.998	0.178	0.562	0.939	0.174	0.637	0.205
	*T*_*Risk*_	0.022	0.023	0.030	0.026	0.017	0.143	0.502	0.857	0.984	0.128	0.475	0.884	0.129	0.497	0.112
SMLE	*T*_*MO*_	0.024	0.031	0.036	0.031	0.023	0.163	0.587	0.926	0.999	0.154	0.536	0.929	0.151	0.598	0.167
	*T*_*MC*_	0.053	0.048	0.043	0.047	0.052	0.283	0.682	0.946	0.999	0.184	0.566	0.947	0.180	0.690	0.285
	*T*_*ML*_	0.048	0.045	0.040	0.044	0.049	0.266	0.662	0.938	0.998	0.174	0.551	0.941	0.171	0.672	0.270

	*T*_*MW*_	0.029	0.040	0.044	0.040	0.028	0.191	0.632	0.940	0.999	0.180	0.572	0.941	0.178	0.644	0.198
	*T*_*Risk*_	0.011	0.018	0.032	0.026	0.018	0.085	0.448	0.864	0.994	0.120	0.490	0.906	0.141	0.547	0.134
DBCD	*T*_*MO*_	0.026	0.033	0.042	0.031	0.024	0.178	0.609	0.934	0.999	0.165	0.555	0.933	0.161	0.619	0.185
	*T*_*MC*_	0.052	0.046	0.045	0.046	0.048	0.280	0.688	0.948	0.999	0.185	0.573	0.949	0.181	0.696	0.284
	*T*_*ML*_	0.040	0.043	0.043	0.043	0.038	0.244	0.667	0.945	0.999	0.178	0.565	0.944	0.174	0.680	0.252

	*T*_*MW*_	0.032	0.041	0.043	0.037	0.030	0.207	0.647	0.935	0.996	0.183	0.562	0.924	0.186	0.636	0.204
	*T*_*Risk*_	0.047	0.040	0.035	0.032	0.028	0.214	0.605	0.903	0.993	0.152	0.503	0.894	0.140	0.528	0.146
SEU	*T*_*MO*_	0.014	0.026	0.032	0.023	0.020	0.127	0.540	0.900	0.995	0.148	0.520	0.914	0.150	0.587	0.159
	*T*_*MC*_	0.049	0.047	0.043	0.047	0.052	0.268	0.676	0.938	0.998	0.187	0.564	0.945	0.191	0.695	0.284
	*T*_*ML*_	0.059	0.049	0.042	0.044	0.049	0.285	0.677	0.935	0.995	0.182	0.551	0.922	0.183	0.665	0.268

	*T*_*MW*_	0.029	0.037	0.049	0.041	0.030	0.203	0.657	0.943	0.999	0.167	0.573	0.929	0.178	0.617	0.192
	*T*_*Risk*_	0.024	0.032	0.046	0.035	0.031	0.183	0.625	0.936	0.999	0.158	0.560	0.922	0.165	0.583	0.166
GDL	*T*_*MO*_	0.013	0.026	0.043	0.034	0.033	0.124	0.587	0.930	0.999	0.150	0.552	0.928	0.161	0.619	0.204
	*T*_*MC*_	0.051	0.047	0.050	0.050	0.058	0.281	0.700	0.948	0.999	0.177	0.579	0.949	0.187	0.695	0.298
	*T*_*ML*_	0.050	0.047	0.046	0.039	0.043	0.282	0.700	0.947	0.999	0.176	0.563	0.933	0.169	0.652	0.258

**Table 10:** Power and type I error at *R*_*LLR *_(alpha = 0.05, *n *= 30).

*p*_1_		0.200	0.300	0.500	0.700	0.800	0.100	0.100	0.100	0.100	0.300	0.300	0.300	0.500	0.500	0.700
*p*_2_		0.200	0.300	0.500	0.700	0.800	0.300	0.500	0.700	0.900	0.500	0.700	0.900	0.700	0.900	0.900
	*T*_*MW*_	0.034	0.043	0.046	0.044	0.031	0.212	0.659	0.946	0.999	0.187	0.575	0.948	0.182	0.667	0.218
	*T*_*Risk*_	0.039	0.034	0.033	0.022	0.008	0.203	0.597	0.911	0.995	0.146	0.490	0.869	0.124	0.432	0.072
SMLE	*T*_*MO*_	0.018	0.029	0.040	0.031	0.017	0.129	0.577	0.931	0.999	0.162	0.549	0.934	0.156	0.587	0.133
	*T*_*MC*_	0.052	0.050	0.046	0.052	0.051	0.274	0.692	0.951	0.999	0.192	0.578	0.953	0.185	0.700	0.278
	*T*_*ML*_	0.060	0.050	0.044	0.051	0.057	0.289	0.691	0.948	0.999	0.186	0.567	0.950	0.181	0.698	0.289

	*T*_*MW*_	0.036	0.047	0.050	0.045	0.031	0.223	0.688	0.957	0.999	0.192	0.591	0.956	0.192	0.697	0.225
	*T*_*Risk*_	0.063	0.049	0.037	0.012	0.001	0.278	0.686	0.947	0.998	0.171	0.528	0.872	0.129	0.356	0.026
DBCD	*T*_*MO*_	0.010	0.028	0.046	0.026	0.009	0.094	0.569	0.946	0.999	0.169	0.579	0.942	0.171	0.580	0.094
	*T*_*MC*_	0.050	0.055	0.051	0.052	0.044	0.265	0.710	0.959	0.999	0.197	0.592	0.959	0.197	0.715	0.267
	*T*_*ML*_	0.071	0.062	0.051	0.057	0.066	0.315	0.727	0.960	0.999	0.198	0.591	0.959	0.199	0.733	0.316

	*T*_*MW*_	0.034	0.043	0.046	0.043	0.033	0.215	0.665	0.947	0.999	0.187	0.581	0.947	0.186	0.671	0.214
	*T*_*Risk*_	0.047	0.038	0.031	0.018	0.007	0.226	0.617	0.915	0.995	0.148	0.492	0.854	0.125	0.414	0.063
SEU	*T*_*MO*_	0.016	0.027	0.038	0.028	0.013	0.124	0.573	0.931	0.999	0.161	0.553	0.929	0.157	0.574	0.123
	*T*_*MC*_	0.052	0.049	0.047	0.050	0.050	0.276	0.696	0.952	0.999	0.191	0.583	0.951	0.191	0.701	0.270
	*T*_*ML*_	0.063	0.051	0.044	0.052	0.061	0.294	0.696	0.949	0.999	0.186	0.573	0.948	0.186	0.701	0.292

	*T*_*MW*_	0.033	0.037	0.043	0.038	0.032	0.230	0.670	0.950	1.000	0.178	0.585	0.956	0.177	0.675	0.215
	*T*_*Risk*_	0.035	0.032	0.036	0.018	0.005	0.230	0.645	0.937	0.999	0.151	0.537	0.905	0.139	0.449	0.049
GDL	*T*_*MO*_	0.016	0.030	0.043	0.031	0.014	0.139	0.614	0.945	1.000	0.172	0.582	0.951	0.172	0.612	0.127
	*T*_*MC*_	0.052	0.050	0.044	0.048	0.053	0.293	0.719	0.955	1.000	0.189	0.588	0.960	0.186	0.722	0.275
	*T*_*ML*_	0.063	0.051	0.044	0.049	0.064	0.322	0.722	0.955	1.000	0.189	0.587	0.960	0.187	0.728	0.302

**Table 11:** Power and type I error at *R*_*RSIHR *_(alpha = 0.05, *n *= 30).

*p*_1_		0.200	0.300	0.500	0.700	0.800	0.100	0.100	0.100	0.100	0.300	0.300	0.300	0.500	0.500	0.700
*p*_2_		0.200	0.300	0.500	0.700	0.800	0.300	0.500	0.700	0.900	0.500	0.700	0.900	0.700	0.900	0.900
	*T*_*MW*_	0.028	0.045	0.056	0.048	0.035	0.174	0.648	0.944	0.999	0.192	0.588	0.946	0.202	0.678	0.228
	*T*_*Risk*_	0.118	0.085	0.058	0.034	0.018	0.343	0.712	0.950	0.999	0.207	0.568	0.910	0.172	0.515	0.102
SMLE	*T*_*MO*_	0.004	0.012	0.040	0.034	0.023	0.037	0.397	0.890	0.998	0.130	0.538	0.936	0.170	0.616	0.156
	*T*_*MC*_	0.038	0.049	0.056	0.057	0.057	0.200	0.657	0.945	0.999	0.192	0.591	0.953	0.208	0.718	0.290
	*T*_*ML*_	0.070	0.065	0.056	0.054	0.062	0.291	0.685	0.945	0.998	0.196	0.579	0.946	0.197	0.705	0.301

	*T*_*MW*_	0.020	0.050	0.057	0.050	0.038	0.157	0.654	0.948	0.999	0.201	0.605	0.956	0.217	0.700	0.242
	*T*_*Risk*_	0.138	0.103	0.062	0.030	0.013	0.383	0.732	0.953	0.999	0.227	0.594	0.922	0.186	0.534	0.097
DBCD	*T*_*MO*_	0.001	0.007	0.038	0.034	0.020	0.017	0.323	0.887	0.999	0.123	0.554	0.942	0.185	0.628	0.159
	*T*_*MC*_	0.028	0.056	0.057	0.057	0.060	0.183	0.662	0.948	0.999	0.202	0.607	0.959	0.221	0.733	0.304
	*T*_*ML*_	0.074	0.079	0.057	0.052	0.064	0.293	0.693	0.948	0.999	0.208	0.593	0.954	0.207	0.726	0.317

	*T*_*MW*_	0.029	0.039	0.050	0.044	0.033	0.181	0.626	0.930	0.998	0.178	0.559	0.932	0.182	0.653	0.214
	*T*_*Risk*_	0.095	0.070	0.044	0.024	0.010	0.275	0.650	0.926	0.996	0.163	0.512	0.875	0.137	0.449	0.071
SEU	*T*_*MO*_	0.014	0.021	0.037	0.028	0.016	0.075	0.466	0.892	0.997	0.137	0.521	0.921	0.152	0.574	0.128
	*T*_*MC*_	0.044	0.045	0.050	0.053	0.049	0.225	0.642	0.932	0.998	0.181	0.562	0.945	0.189	0.696	0.271
	*T*_*ML*_	0.058	0.053	0.050	0.052	0.062	0.268	0.657	0.929	0.997	0.178	0.548	0.934	0.179	0.684	0.289

	*T*_*MW*_	0.031	0.048	0.052	0.050	0.036	0.206	0.682	0.951	1.000	0.197	0.610	0.961	0.212	0.690	0.235
	*T*_*Risk*_	0.084	0.065	0.050	0.026	0.009	0.321	0.715	0.952	1.000	0.201	0.591	0.919	0.173	0.495	0.076
GDL	*T*_*MO*_	0.002	0.016	0.042	0.034	0.017	0.047	0.476	0.923	1.000	0.147	0.577	0.947	0.186	0.613	0.142
	*T*_*MC*_	0.040	0.052	0.052	0.056	0.053	0.228	0.689	0.952	1.000	0.198	0.611	0.964	0.216	0.721	0.289
	*T*_*ML*_	0.074	0.062	0.051	0.055	0.063	0.301	0.707	0.952	1.000	0.199	0.602	0.962	0.207	0.722	0.316

**Table 12:** Power and type I error at *R*_*RPW *_(alpha = 0.05, *n *= 30).

*p*_1_		0.200	0.300	0.500	0.700	0.800	0.100	0.100	0.100	0.100	0.300	0.300	0.300	0.500	0.500	0.700
*p*_2_		0.200	0.300	0.500	0.700	0.800	0.300	0.500	0.700	0.900	0.500	0.700	0.900	0.700	0.900	0.900
	*T*_*MW*_	0.031	0.039	0.050	0.050	0.042	0.191	0.631	0.918	0.966	0.166	0.538	0.859	0.183	0.585	0.204
	*T*_*Risk*_	0.071	0.058	0.059	0.061	0.060	0.287	0.683	0.939	0.993	0.193	0.565	0.905	0.197	0.607	0.216
RPW	*T*_*MO*_	0.004	0.012	0.032	0.038	0.039	0.047	0.410	0.840	0.967	0.105	0.467	0.867	0.151	0.584	0.196
	*T*_*MC*_	0.045	0.042	0.050	0.063	0.075	0.227	0.640	0.921	0.988	0.167	0.546	0.914	0.196	0.680	0.301
	*T*_*ML*_	0.067	0.050	0.049	0.049	0.053	0.288	0.661	0.916	0.931	0.172	0.523	0.820	0.173	0.573	0.235

	*T*_*MW*_	0.032	0.043	0.052	0.050	0.040	0.208	0.658	0.944	0.998	0.183	0.586	0.939	0.204	0.658	0.219
	*T*_*Risk*_	0.057	0.051	0.055	0.048	0.032	0.273	0.679	0.947	0.998	0.192	0.588	0.935	0.199	0.612	0.164
DL	*T*_*MO*_	0.003	0.013	0.038	0.041	0.033	0.047	0.464	0.906	0.998	0.123	0.527	0.934	0.172	0.641	0.193
	*T*_*MC*_	0.043	0.045	0.052	0.062	0.064	0.237	0.662	0.944	0.999	0.184	0.592	0.956	0.216	0.723	0.307
	*T*_*ML*_	0.058	0.050	0.050	0.049	0.056	0.275	0.672	0.943	0.998	0.183	0.567	0.940	0.188	0.688	0.283

	*T*_*MW*_	0.027	0.040	0.048	0.049	0.044	0.188	0.626	0.921	0.968	0.167	0.537	0.848	0.175	0.550	0.195
	*T*_*Risk*_	0.073	0.062	0.058	0.063	0.072	0.283	0.678	0.936	0.993	0.193	0.563	0.910	0.196	0.617	0.247
SMLE	*T*_*MO*_	0.006	0.012	0.031	0.040	0.049	0.054	0.409	0.840	0.969	0.108	0.463	0.864	0.148	0.584	0.229
	*T*_*MC*_	0.039	0.044	0.049	0.061	0.079	0.226	0.636	0.922	0.989	0.168	0.547	0.911	0.190	0.671	0.315
	*T*_*ML*_	0.064	0.054	0.046	0.046	0.047	0.287	0.659	0.917	0.925	0.171	0.519	0.794	0.165	0.528	0.200

	*T*_*MW*_	0.031	0.037	0.053	0.049	0.044	0.202	0.635	0.929	0.969	0.181	0.529	0.813	0.173	0.503	0.192
	*T*_*Risk*_	0.063	0.054	0.065	0.072	0.081	0.290	0.685	0.942	0.994	0.202	0.572	0.911	0.209	0.640	0.285
DBCD	*T*_*MO*_	0.003	0.010	0.033	0.043	0.054	0.041	0.407	0.866	0.981	0.110	0.460	0.856	0.146	0.573	0.257
	*T*_*MC*_	0.041	0.040	0.054	0.067	0.083	0.236	0.640	0.930	0.990	0.181	0.543	0.905	0.195	0.660	0.325
	*T*_*ML*_	0.061	0.048	0.052	0.042	0.036	0.289	0.661	0.925	0.857	0.183	0.511	0.696	0.160	0.407	0.144

	*T*_*MW*_	0.033	0.040	0.047	0.041	0.032	0.204	0.633	0.924	0.994	0.183	0.553	0.908	0.185	0.618	0.199
	*T*_*Risk*_	0.076	0.059	0.058	0.048	0.043	0.278	0.664	0.929	0.996	0.183	0.529	0.899	0.170	0.564	0.182
SEU	*T*_*MO*_	0.012	0.021	0.028	0.027	0.024	0.100	0.467	0.855	0.993	0.130	0.493	0.900	0.143	0.578	0.169
	*T*_*MC*_	0.051	0.047	0.050	0.059	0.065	0.251	0.652	0.925	0.997	0.186	0.556	0.933	0.197	0.686	0.286
	*T*_*ML*_	0.062	0.051	0.048	0.047	0.049	0.293	0.671	0.923	0.992	0.185	0.541	0.904	0.183	0.642	0.251

	*T*_*MW*_	0.032	0.045	0.049	0.045	0.032	0.216	0.658	0.937	0.998	0.171	0.576	0.916	0.192	0.602	0.196
	*T*_*Risk*_	0.056	0.053	0.053	0.050	0.042	0.281	0.681	0.942	0.998	0.180	0.586	0.927	0.196	0.615	0.197
GDL	*T*_*MO*_	0.004	0.017	0.036	0.040	0.037	0.066	0.480	0.900	0.998	0.122	0.525	0.918	0.165	0.622	0.219
	*T*_*MC*_	0.044	0.049	0.050	0.058	0.061	0.250	0.666	0.939	0.999	0.173	0.584	0.948	0.206	0.700	0.314
	*T*_*ML*_	0.061	0.054	0.047	0.044	0.043	0.294	0.681	0.937	0.998	0.175	0.560	0.920	0.179	0.639	0.256

As shown in Table 6 (also see Tables 7, 8, 9, 10, 11, 12), the worst performance can be found in the results of *T*_*MO *_and *T*_*Risk*_, which are often conservative with less than nominal type I error rate. *T*_*MW *_is always slightly conservative across all simulation cases. Overall, *T*_*MC *_is the best in attaining the correct type I error rate. *T*_*ML*_, is slightly inflated as compared with chi-square test *T*_*MC*_. However, the simulation results not shown here indicate that *T*_*ML *_is very robust against the unbalance in patient allocation even when sample size is 20. The comparison between different RAR methods shows that the mean type I error of GDL and SEU can usually match the correct size of tests better than other methods when *T*_*MC *_and *T*_*ML *_are used respectively. The type I error of DBCD is usually the largest one, except at *R*_*Odds*_. The overall type I error of SEU is comparable with GDL.

The power comparison of different test statistics indicates that *T*_*Risk *_is the statistic with the highest power at *R*_*Risk *_but with a much inflated type I error. Except at *R*_*Risk*_, *T*_*MC *_or *T*_*ML *_is the one with the highest power. Usually, GDL has the highest power and SEU has the lowest power among all RAR methods. DBCD and SMLE have similar power, but DBCD is more powerful in most cases. At target *R*_*RPW*_, DL urn has the best statistical properties. On the average, the target with the lowest power achieved by test statistics is *R*_*Risk*_. The highest overall power can usually be achieved by test statistics at *R*_*RSIHR *_and *R*_*LLR*_, but *R*_*LLR *_has the disadvantage of assigning more patients to the worse treatment in some cases.

## Discussion

In response-adaptive randomization, the assignment of a new patient depends on the treatment outcomes of patients previously enrolled in the trial. Delayed responses are often encountered in practice. Recently, the problem of delayed response in multi-arm generalized drop-the-loser urn and generalized Friedman's urn design is studied for both continuous and discontinuous outcomes [11,16,17,36]. It is shown that, under reasonable assumption about the delay, the asymptotic properties of adaptive design are not affected by the delay. In the present study, the primary focus is the comparison between commonly used test statistics for 2 × 2 tables. Based on results not shown here, a less extreme allocation with higher variation would be expected when a random delay is assumed. It is assumed that the response status of each of the patients already in the trial is available before the allocation of a new patient in our simulations evaluation.

The RAR methods simulated in the present study are aimed at assigning patients to the better treatment with probabilities higher than what otherwise would be allowed by equal randomization. The price being paid is that the sample sizes on the two comparing arms are no longer fixed, and the adaptation in patient allocation can complicate the statistical inference at the end of the trial. The properties of test statistics will change when the patient allocation ratio changes in adaptive randomization. The power of test statistics shown in the present simulation study is obtained by averaging over trials with an unknown distribution of allocation ratios. As shown in our simulation results, a large deviation from the nominal significance level of the hypothesis test can be found even under the null hypothesis. Therefore, the practice of comparing asymptotic hypothesis testing methods based solely on statistical power under the alternative hypothesis is not recommended. It is important to compare adaptive randomization methods based on both the type I error rate and the statistical power, especially when the sample size is small.

General recommendations given in the result section are based on the aggregated results across different settings. Because the performance of different test statistics, RAR methods, and allocation target are closely related to each other, recommendations under a specific scenario can be found based on the detailed simulation results in Tables 7, 8, 9, 10, 11, 12.

Based on simulation results, the Cook's correction to chi-square test statistic *T*_*MC *_and Williams' correction to log-likelihood-ratio test *T*_*ML *_are recommended to be used for hypothesis testing at the end of adaptive randomization. *T*_*MC *_has good ability to attain the correct significance levels, and is relatively robust against the change of RAR method or allocation target. *T*_*ML *_has more robust performance than *T*_*MC *_and has higher power, but its type I error is slightly inflated as compared with *T*_*MC*_. However, *T*_*ML *_attains more accurate type I error than *T*_*MC *_when the sample size is small. The original Wald-type Z test statistic *T*_*Wald*_, which is very sensitive to patient allocation and has inflated type I error, should be avoided at small sample sizes. On the other hand, *T*_*MW*_, the Argresti's correction to *T*_*Wald*_, and *T*_*MO *_the modified log-odds-ratio test are too conservative and under powered at small sample sizes.

The primary objective of current study is to compare test statistics. Since the recommended test statistics are *T*_*MC *_and *T*_*ML*_, the comparison between RAR methods and allocation targets are mainly based on these two selected test statistics. Among SMLE, DBCD, SEU, and GDL methods, GDL seems to be the best one due to its ability to attain the correct size of hypothesis test and comparatively higher overall power at most allocation targets. Therefore, GDL is the recommended RAR method. The sequential estimation-adjusted urn (SEU) method is comparable with GDL in controlling the type I error. However, SEU is often under powered, and the high variation in patient allocation makes it less useful in practice. The DBCD method with tuning exponent *α *equal to 2 is the best in targeting the true allocation ratio. When *T*_*MC *_is the test statistic, DBCD has slightly inflated type I error and slightly lower power as compared with GDL. Therefore, among values of *α*, the balances among controlling the type I error, obtaining higher power, and targeting a given allocation ratio can be reached when *α *is equal to 2. The simulation comparison of statistical power for different RAR methods also indicates that DL urn has the best statistical properties at *R*_*RPW*_, mainly due to its low variation in patient allocation.

The statistical characteristics of hypothesis tests and RAR methods also depend on allocation targets. At *R*_*Wald*_, *R*_*Odds*_, and *R*_*LLR *_targets, more patients could be assigned to the inferior treatment in certain parameter spaces. In contrast, *R*_*Risk*_, *R*_*RPW*_, and *R*_*RSIHR *_always assign more patients to the better treatment. However, due to the more extreme allocation of *R*_*Risk *_and *R*_*RPW*_, both power and type I error of *R*_*Risk *_and *R*_*RPW *_will suffer as compared with *R*_*RSIHR*_. On the other hand, the variation of patient allocation at *R*_*RISHR *_is relatively small with a stable value across all simulation scenarios. Additional, among all designs with similar power using Wald-type test statistic, *R*_*RSIHR *_allocation ration can achieve fewer failures in the whole trial. Therefore, *R*_*RSIHR *_is recommended among all the allocation targets in the present study.

In addition to the frequentist development on the response adaptive randomization, Bayesian decision theoretic methods has also been proposed in the context of bandit problem. The concept of "patient horizon" was brought up to include future patients to whom the current study results might be applied. The goal is to maximize the total number of success in patients enrolled in the study with or without including the patient horizon. More detailed exposition of Bayesian methods for response adaptive randomization is beyond the scope of this paper and interested readers should consult the original work on this topic [37-40].

## Conclusion

The Cook's correction to chi-square test and Williams' correction to log-likelihood-ratio test are recommended for hypothesis test of RAR at small sample sizes. Among all the RAR methods compared, GDL method has better statistical properties in controlling type one error and maintaining high statistical power. The RSIHR allocation target provides a good balance between assigning more patients to the better treatment and maintaining a high overall power.

## Abbreviations

RAR: Response-adaptive randomization; RPW: Randomized play-the-winner; DL: Drop-the-loser; DBCD: Doubly-adaptive biased coin design; SMLE: Sequential maximum likelihood estimation design; SEU: Sequential estimation-adjusted urn; GDL: Generalized drop-the-loser urn; RSIHR: Optimal allocation target minimizing total numbers of failure for Wald-type test statistics at fixed power; MLE: Maximum likelihood estimate.

## Competing interests

The authors declare that they have no competing interests.

## Authors' contributions

XMG conducted the simulation part of the study. Both XMG and JJL participated in designing the study and writing the manuscript. All authors read and approved the final manuscript.

## Pre-publication history

The pre-publication history for this paper can be accessed here:

http://www.biomedcentral.com/1471-2288/10/48/prepub
